# Terahertz High-Sensitivity SPR Phase Biosensor Based on the Weyl Semimetals

**DOI:** 10.3390/bios15090606

**Published:** 2025-09-15

**Authors:** Yu Xie, Zean Shen, Mengjiao Ren, Mingming Zhang, Mingwei Guo, Leyong Jiang

**Affiliations:** 1 School of Physics and Electronics, Hunan Normal University, Changsha 410081, China; xieyu@hunnu.edu.cn (Y.X.); shenzean@hunnu.edu.cn (Z.S.); renmengjiao@hunnu.edu.cn (M.R.); zhangmingming@hunnu.edu.cn (M.Z.); 2 Hunan Provincial Key Laboratory of Post-Molar Physics and Devices, Hunan Normal University, Changsha 410081, China

**Keywords:** terahertz biosensor, surface plasmon resonance, weyl semimetals, deep learning

## Abstract

Optical biosensors play a crucial role in the field of biological detection by converting biological signals into optical signals for detection. Among them, Surface Plasmon Resonance (SPR) optical biosensors have become a research hotspot in this field due to their significant advantage of high sensitivity. Weyl Semimetals (WSMs), as a type of three-dimensional topological material with unique electronic structures and other properties, exhibit potential applications in the field of SPR sensing. Against this background, we designed a terahertz (THz) high-sensitivity SPR phase biosensor with a KR structure based on WSMs. When applied in gas sensing scenarios, the phase detection sensitivity of this sensor can reach 22,402°/RIU, showing a significant improvement compared to traditional SPR biosensors. Moreover, we found that the Weyl node separation distance and twist angle of WSMs have obvious effects on sensitivity regulation. Additionally, we optimized the sensitivity and structural parameters of this structure using a neural network-based deep learning algorithm. We expect that this proposed scheme can provide a feasible reference for the field of biological sensing.

## 1. Introduction

As a pivotal technological tool in the field of biosensing, optical biosensors possess significant advantages in this domain. They are capable of converting certain biological signals that are difficult to measure into optical signals that are easy to capture and quantify [1]. Endowed with characteristics such as non-contact measurement, non-invasive detection, and high sensitivity [2,3], these sensors find extensive applications in areas including pathogenic microorganism detection [4,5], small biological molecule analysis [6], and drug testing [7,8]. In recent years, with the development of micro- and nano-processing technologies, micro- and nano-scale biosensors have played a vital role in rapid, real-time detection of biomolecules due to their miniaturization and easy integration [9]. Currently, optical biosensors based on structures such as micro-ring resonators [10], optical waveguide [11], plasmonic nanostructures [12,13], terahertz (THz) plasmonic [14], and gratings [15] have been widely reported. Surface plasmon resonance (SPR) biosensors are highly sensitive to changes in the surrounding environment and exhibit excellent sensitivity, thus receiving long-term attention in the field of biosensing [16]. For instance, Herranz et al. developed an SPR biosensor for detecting microcystin-LR in drinking water, which enables four simultaneous measurements within 60 min [17]. In particular, SPR is extremely sensitive to variations in the refractive index of the surrounding medium; even tiny changes in the refractive index of the sensing medium can induce a shift in the resonance angle of the SPR reflectivity curve, thereby enabling the detection of biomolecules [18]. However, traditional SPR biosensors, typically represented by metals, have certain limitations in the detection of small-molecule compounds and ultra-low concentration analytes. Particularly in the THz band, they suffer from issues such as attenuated SPR excitation efficiency and the lack of dynamic tunability, which in turn restrict the development of such sensors [19,20].

Over the past few years, two-dimensional materials, represented by graphene [21], black phosphorus [22], and MXene [23], have opened up new avenues for the research of novel SPR optical biosensors, thanks to their exceptional and unique optoelectronic properties. For instance, Maharana et al. excited SPR by coating a single layer of graphene on a silver surface. Different from biosensors using a gold-silver bimetallic combination, this design exhibits higher sensitivity and a narrower full width at half maximum (FWHM) [24]; Meshginqalam et al. designed an SPR biosensor on the basis of the BP/WS_2_ structure, whose sensitivity is more than twice that of traditional sensors, reaching a maximum of 187°/RIU [25]; Zeng et al. introduced MoS_2_ into a graphene-based SPR biosensor to develop a new type of SPR biosensor that is reliable and possesses excellent performance [26]. Similarly, the emergence of the Weyl semimetals (WSMs) has provided new options for the design and realization of novel optical biosensors. WSMs have shown potential applications in optical biosensing due to their unique electronic structure [27,28]. WSMs can effectively excite SPR under specific conditions [29]. Unlike traditional SPR sensors, which mostly rely on refractive index variation for detection, WSMs possess anisotropy; by detecting the phase change of light, they demonstrate higher sensitivity in responding to intermolecular interactions [30]. Notably, the quasiparticles of WSMs at specific electronic band crossing points (Weyl nodes) exhibit fermion properties described by the Weyl quantum field equation, allowing WSMs to be regarded as three-dimensional "graphene-like" materials [31]. What is more, in comparison with other two-dimensional materials, WSMs benefit from high electron mobility and linear energy band dispersion near Weyl nodes, which endow them with a more stable three-dimensional topological structure. This unique structure makes WSMs promising for achieving stronger light-matter interactions in the THz range [32]. Additionally, WSMs possess topologically protected properties, which render them less susceptible to interference such as external noise. This protection allows light to propagate unimpeded along designated paths with minimal loss [33]. It is thus evident that WSMs can provide new insights for the design of tunable SPR phase sensors [34].

Against this backdrop, this study designs a THz high-sensitivity SPR phase biosensor with a KR [35] structure based on WSMs. The sensor combines a coupling prism with WSMs to excite SPR, which lays a foundation for achieving higher phase sensitivity. Meanwhile, the Weyl node separation distance and the twist angle of WSMs also provide means for realizing dynamic regulation. We profoundly analyzed the sensor’s performance in the THz band and applied it to gas sensing scenarios. By optimizing the two aforementioned parameters, the phase detection sensitivity of this sensor can reach 22,402°/RIU. In addition, we optimized the sensor structure and its parameters in accordance with forward modeling and inverse design enabled by deep learning. We truly hope this proposed scheme holds potential applications in the field of biosensing.

## 2. Theoretical Model and Method

**Figure 1:**
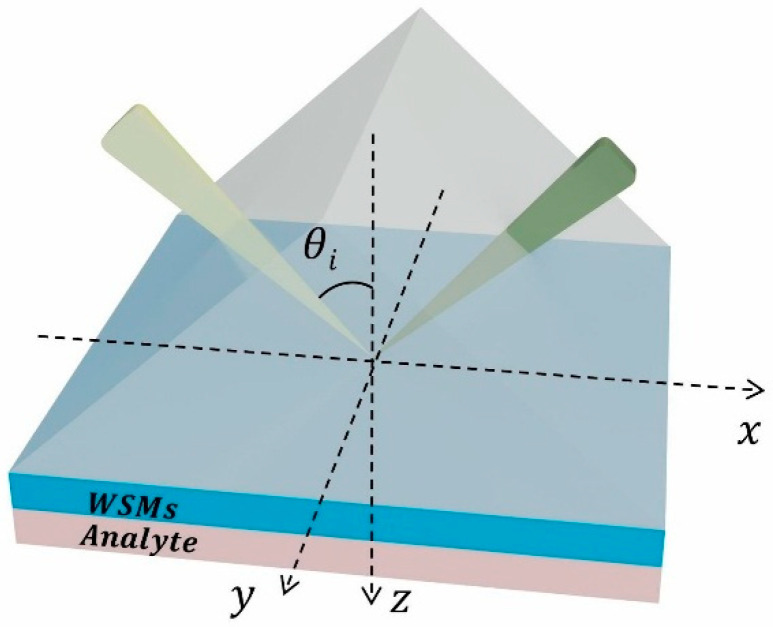
Schematic structure of THz high-sensitivity SPR phase biosensor based on WSMs.

We consider a KR structure based on WSMs. From top to bottom, this structure consists of a coupling prism, a WSMs layer, and an air sensing layer, as illustrated in Figure 1. Specifically, the coupling prism is made of FK51A material with a dielectric constant of $\varepsilon_{Fk51A}=2.6$ [36]. The photoelectric properties of WSMs are characterized using a bulk medium model, and their volume conductivity can be expressed as follows [31]:

(1) $$\sigma =\frac{gr_{s}}{6}\Omega G\left(\frac{\hbar \Omega }{2}\right)+i\frac{gr_{s}}{6\pi }\left\{\frac{4}{\hbar^{2}\Omega }\left[E_{F}^{2}+\frac{\pi^{2}}{3}{\left(k_{B}T\right)}^{2}\right]+8\Omega \int_{0}^{E_{c}}\frac{G(E)-G\left(\frac{\hbar \Omega }{2}\right)}{{(\hbar \Omega )\;}^{2}-4E^{2}}EdE\right\},$$

Among these parameters, $r_{s}=e^{2}/(4\pi \varepsilon_{0}\hbar v_{F})$ is a constant with Fermi velocity, $\Omega =\omega +i\tau^{-1}$ denotes the complex frequency; $\tau^{-1}$ represents the scattering rate of Drude damping, n(E) stands for the Fermi distribution; G(E)=n(−E)−n(E), g is the number of Weyl nodes in WSMs; *E_F_(T)* is the chemical potential dependent on temperature (*T_a_*) and stopping potential (*E_C_*), which can be expressed by the following formula:

(2) $$E_{F}(T_{a})=\frac{2^{1/3}{\left[9E_{F}{\left(0\right)}^{3}+\sqrt{81E_{F}{\left(0\right)}^{6}+12\pi^{6}k_{B}^{6}{T_{a}}^{6}}\right]}^{2/3}-2\pi^{2}3^{1/3}k_{B}^{2}{T_{a}}^{2}}{6^{2/3}{\left[9E_{F}{\left(0\right)}^{3}+\sqrt{81E_{F}{\left(0\right)}^{6}+12\pi^{6}k_{B}^{6}{T_{a}}^{6}}\right]}^{1/3}},$$

In this paper, $K_{B}$ is the Boltzmann’s constant, $E_{F}(0)=1.63\;\mathrm{eV}$, $E_{F}(300\;K)=0.150\;\mathrm{eV}$ and the other parameters are set as follows: $\varepsilon_{b}=6.2$, $T_{a}=300\;K$, $E_{c}=3E_{F}$, g=2, $v_{F}=0.83\times 10^{5}\;m/s$, τ=1000fs, $E_{F}=0.15$ eV. In calculations related to sensing, the Transmission Matrix Method (TMM) is typically employed to compute the reflectance (*R*) and transmittance (*T*) of multilayer structures. In this study, since anisotropic media are involved, we calculate *R* and *T* based on the generalized Fresnel equations combined with the 4 × 4 transmission matrix formulation [37]. The transmission matrix for each layer is denoted as $T_{i}=A_{i}P_{i}A_{i}^{-1}$, the total transmission matrix is $T_{tot}=\prod_{i=1}^{N}T_{i}$, and the transmission matrix for the entire structure, including the first dielectric layer and the last dielectric layer, can be expressed as follows [38,39]:

(3) $$T_{N}=A_{0}^{-1}T_{tot}A_{N+1}=A_{0}T_{1}T_{2}\ldots T_{N}A_{N+1}=L_{1}P_{1}L_{2}P_{2}\ldots L_{N}P_{N}L_{N+1},$$

We map the transmission matrix to the electric field components in a one-to-one correspondence and perform the transformation. The transformed transmission matrix can be expressed as follows:

(4) $$T_{N}^{*}={\left(\begin{array}{cccc} 1 & 0 & 0 & 0\\ 0 & 0 & 1 & 0\\ 0 & 1 & 0 & 0\\ 0 & 0 & 0 & 1 \end{array}\right)}^{-1}T_{N}\left(\begin{array}{cccc} 1 & 0 & 0 & 0\\ 0 & 0 & 1 & 0\\ 0 & 1 & 0 & 0\\ 0 & 0 & 0 & 1 \end{array}\right),$$

From the transformed transmission matrix, the reflection coefficient and transmission coefficient can be obtained, denoted, respectively, as $R_{ps}={\left|r_{ps}\right|}^{2}$, $T_{ps}={\left|t_{ps}\right|}^{2}$, and [40]:

$$\left\{\begin{array}{ll} r_{pp}=\frac{T_{21}^{*}T_{33}^{*}-T_{23}^{*}T_{31}^{*}}{T_{11}^{*}T_{33}^{*}-T_{13}^{*}T_{31}^{*}},\;\;\;t_{pp}=\frac{T_{33}^{*}}{T_{11}^{*}T_{33}^{*}-T_{13}^{*}T_{31}^{*}}; & \left(5a\right)\\ r_{ss}=\frac{T_{11}^{*}T_{43}^{*}-T_{41}^{*}T_{13}^{*}}{T_{11}^{*}T_{33}^{*}-T_{13}^{*}T_{31}^{*}},\;\;\;t_{ss}=\frac{-T_{11}^{*}}{T_{11}^{*}T_{33}^{*}-T_{13}^{*}T_{31}^{*}}; & \left(5b\right)\\ r_{ps}=\frac{T_{41}^{*}T_{33}^{*}-T_{43}^{*}T_{31}^{*}}{T_{11}^{*}T_{33}^{*}-T_{13}^{*}T_{31}^{*}},\;\;\;t_{ss}=\frac{T_{31}^{*}}{T_{11}^{*}T_{33}^{*}-T_{13}^{*}T_{31}^{*}}; & \left(5c\right)\\ r_{sp}=\frac{T_{11}^{*}T_{23}^{*}-T_{21}^{*}T_{13}^{*}}{T_{11}^{*}T_{33}^{*}-T_{13}^{*}T_{31}^{*}},\;\;\;t_{ss}=\frac{T_{13}^{*}}{T_{11}^{*}T_{33}^{*}-T_{13}^{*}T_{31}^{*}}; & \left(5d\right) \end{array}\right.$$

Furthermore, in accordance with the general expression method for sensitivity, we define the phase detection sensitivity as follows:

(6) $$S=\frac{\Delta \varphi }{\Delta n},$$

Among these parameters, Δφ is the differential phase between *p*-polarized light and *s*-polarized light. $\Delta \varphi =\left|\Delta \varphi_{pp}-\Delta \varphi_{ss}\right|$. SPR is excited by *p*-polarized light, while *s*-polarized light is used as a reference signal.

## 3. Results and Discussion

**Figure 2:**
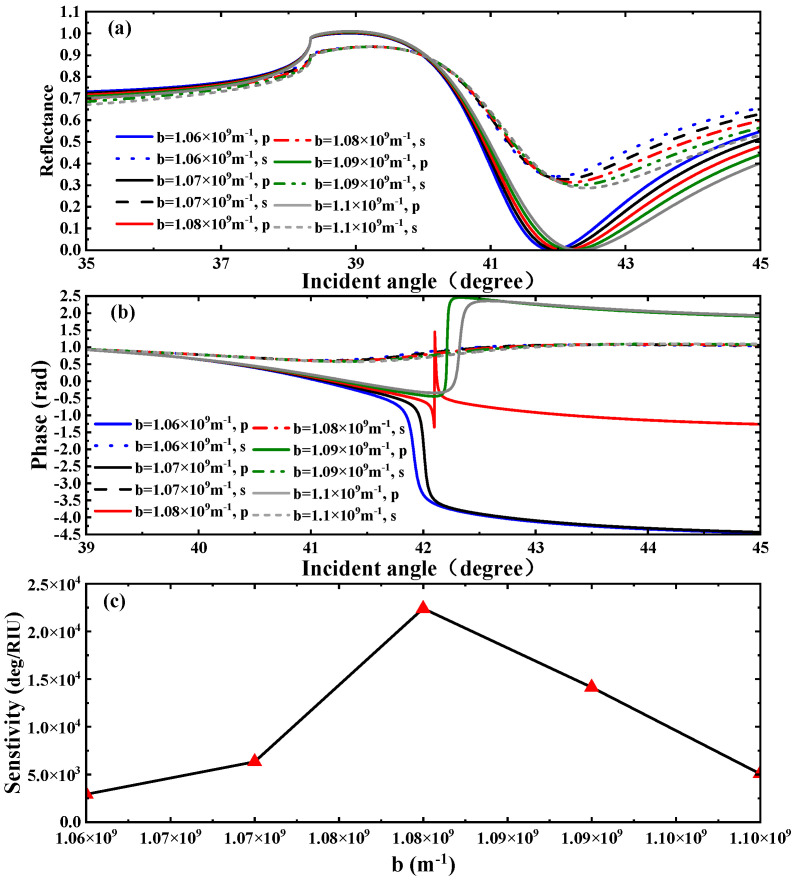
(**a**) SPR curves of reflectance and (**b**) differential phase as a function of Weyl node separation distance. (**c**) Phase detection sensitivities corresponding to different Weyl node separation distance.

In this section, we present a detailed analysis of the sensing performance of the proposed structure. While experimental validation generally entails more practical considerations—particularly those related to micro- and nano-scale fabrication—than theoretical modeling, the current study remains focused on the theoretical aspect, given the relatively complex requirements for experimental realization. It is important to note that well-established experimental methods exist for SPR-based sensing, and the implementation of such experimental work will be the primary focus of our subsequent research phase. First, we focus on investigating the influence of WSMs’ structural parameters on the sensor. WSMs show unique optical properties in the THz band. Given that we select this band as the operating range for WSM materials, the frequency of the incident light is set to 1.7 THz. In the study of WSMs, the Weyl node separation distance *b* and the twist angle *θ* are key parameters of the topological electronic structure [41]. The Weyl node separation distance refers to the separation distance between Weyl nodes in WSMs. By adjusting them, the dielectric response of WSMs can be modified, thereby regulating their optical effects. First, we probe into the influence of the WSMs’ Weyl node separation distance on the reflectance, phase curves, and sensitivity of the SPR spectrum, as shown in Figure 2. We selected five specific values of the Weyl node separation distance to study the excitation of SPR. As expected, this structure can effectively excite SPR under *p*-polarized light. It can be seen from Figure 2a that as the Weyl node separation distance gradually increases, both the reflectance curve and the resonance angle shift to the right. When $b=1.08\times 10^{9}\;m^{-1}$, a relatively low reflectance value of $R_{\min}(2.934\times 10^{-5})$ is obtained. And we also found that the excitation angle of SPR can be effectively controlled by adjusting the Weyl node separat-ion distance. By comparing Figure 2a with Figure 2b, the variation in the phase curve in Figure 2b is more distinct and steeper. Thus, compared with the angular sensitivity scheme, this biosensor is more suitable for the phase detection sensitivity scheme. In particular, when $b=1.08\times 10^{9}\;m^{-1}$, the SPR phase curve exhibits the maximum phase jump at the resonance angle corresponding to the minimum reflectance, as shown in Figure 2b. Calculations using Equation (6) show that the phase detection sensitivity at this point is 22,402°/RIU. In addition, we also analyzed the variation in phase detection sensitivity as the Weyl node separation distance *b* increases from $1.06\times 10^{9}\;m^{-1}$ to $1.10\times 10^{9}\;m^{-1}$. Again, using Equation (6), we calculated, respectively, the corresponding phase detection sensitivities, which are 2922.3°/RIU, 6319.8°/RIU, 22,402°/RIU, 1430°/RIU, and 5064.1°/RIU, as shown in Figure 2c. 

**Figure 3:**
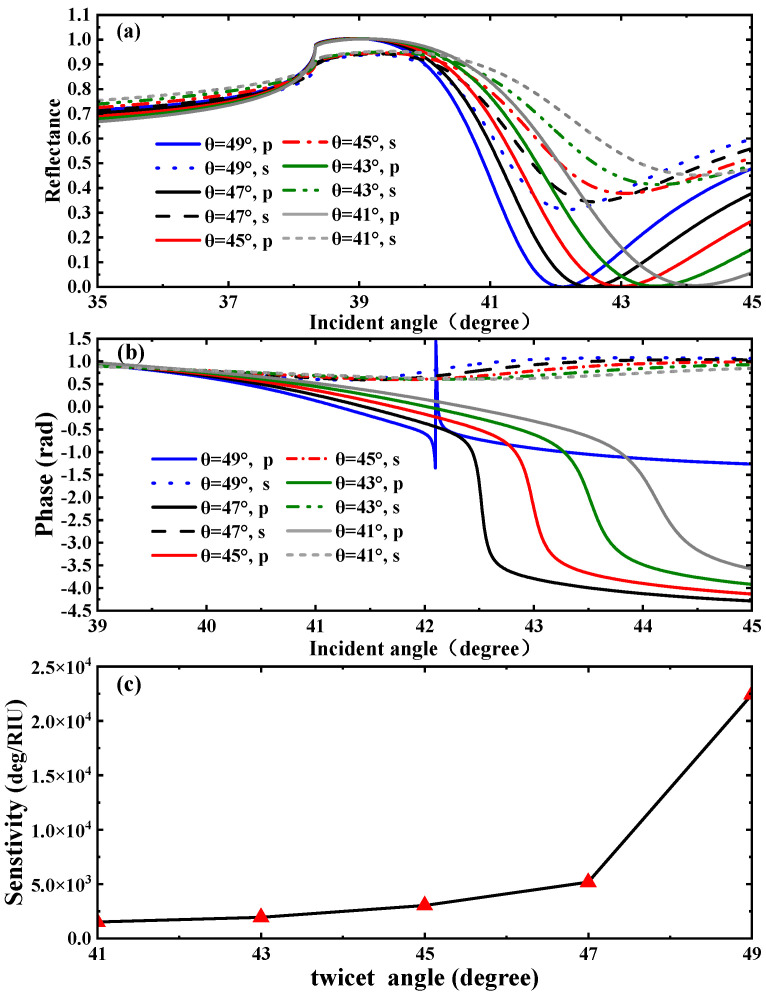
(**a**) SPR curves of reflectance and (**b**) differential phase as a function of twist angle. (**c**) Phase detection sensitivities corresponding to different twist angles.

In the preceding sections, we discussed the influence of varying the Weyl node separation distance of WSM materials on SPR. In general, the excitation and enhancement mechanisms of SPR involve multiple influencing factors, among which the intrinsic properties of the material play a particularly critical role—their regulation effect on the SPR phenomenon cannot be overlooked. By optimizing various parameters of the material, the interaction strength between light and the material can be effectively enhanced, thereby providing strong support for the intensification of the SPR effect. Within the WSMs system, the Weyl node separation distance exerts a significant impact on the band structure of WSMs; changes in the energy band structure, in turn, directly induce corresponding variations in the SPR effect. This underscores the importance of the Weyl node separation distance in the process of regulating SPR using WSMs. Building on this foundation, the angle between the separated wave vectors of the two Weyl nodes in WSMs (i.e., the twist angle θ) is another parameter that deserves focused attention. Variations in the twist angle θ also have a significant regulatory effect on the energy band structure of WSMs—with changes in the twist angle and key characteristics of the energy bands, such as their dispersion relations and energy level distributions, undergoing corresponding adjustments. Such changes in the band structure will inevitably further influence the manifestation of the SPR effect. In light of that, conducting in-depth research on the twist angle holds important theoretical and practical significance for fully understanding the regulatory mechanism of SPR in WSMs. In the meantime, we also analyzed the performance parameters of the sensor based on the influence of the WSMs twist angle on the reflectance, phase curves, and sensitivity of the SPR spectrum, as shown in Figure 3. It can be observed from Figure 3a that as the twist angle gradually decreases, the resonance peak of the SPR curve shifts to the right and the reflection peak gradually broadens. This indicates that as the twist angle decreases, the excited SPR is correspondingly enhanced, reaching the strongest intensity when θ=49°. At this point, a relatively low reflectance $R_{\min}(2.934\times 10^{-5})$ can be acquired. As shown in Figure 3b, the phase of the SPR curve undergoes a phase jump at the resonance angle corresponding to the minimum reflectance. When compared with the reflectance curve in Figure 3a, the variation in the SPR phase curve in Figure 3b is also more apparent. Therefore, we similarly adopt the phase detection sensitivity scheme for calculations. As can be seen from Figure 3b, the SPR biosensor exhibits the maximum phase jump at the twist angle. Calculations using Equation (6) reveal that the phase detection sensitivity at this point is 22,402°/RIU. Likewise, we analyzed the variation in phase sensitivity as the twist angle increases from 41° to 49°. Using Equation (6), the corresponding phase detection sensitivities were calculated, respectively, as 1503.6°/RIU, 1934°/RIU, 3031.5°/RIU, 5164.8°/RIU, 22,402 °/RIU, as shown in Figure 3c. 

**Figure 4:**
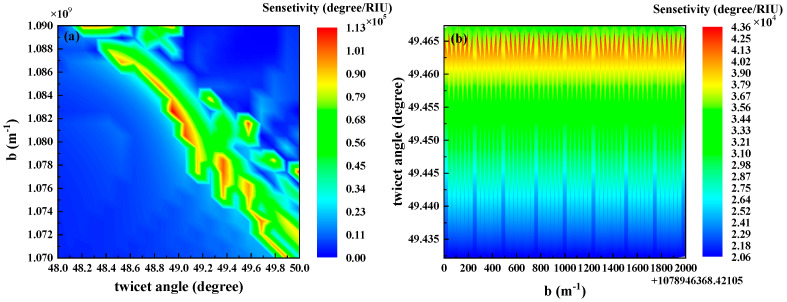
(**a**) Phase sensitivities corresponding to a change in the Weyl node separation distance b from $1.07\times 10^{9}\;m^{-1}$ to $1.09\times 10^{9}\;m^{-1}$ and a change in the twist angles from 48° to 50°. (**b**) Phase sensitivity corresponding to a change in the Weyl node separation distance from $1.078946\times 10^{9}\;m^{-1}$ to $1.078948\times 10^{9}\;m^{-1}$ and a change in the twist angles from 49.435° to 49.465°.

In the research on SPR biosensors based on WSMs, Weyl nodes—unique topological electronic structure features of WSMs—exert a significant influence on the band structure of WSMs through their separation distance. In addition, variations in the twist angle alter key characteristics of WSMs, such as their band dispersion relations and energy level distribution, which in turn affect the excitation efficiency of the SPR effect and sensing performance. In-depth exploration of the regulatory mechanism of Weyl node separation distance and the twist angle on phase detection sensitivity constitutes a critical step in optimizing sensing performance. Based on this, we further studied the combined influence of the Weyl node separation distance and the twist angle on the phase detection sensitivity. Meanwhile, we calculated the relevant phase detection sensitivities when the Weyl node separation distance varies from $1.07\times 10^{9}\;m^{-1}$ to $1.09\times 10^{9}\;m^{-1}$ and the twist angle varies from 49.435° to 49.465°, as shown in Figure 4a. We found that when the twist angle is greater than 48.6°, the phase detection sensitivity varies significantly, and several "information islands" with high sensitivity emerge. These islands indicate that high-sensitivity solution points can be found in certain parameter pairs (b, θ). Among these results, different sensitivities correspond to different parameter combinations, and there are certain regions where the phase sensitivity values are relatively high. To facilitate the subsequent research on the deep learning section, we selected values around the Weyl node separation distance $b=1.0789\;m^{-1}$ and the twist angle θ=49.45°. Specifically, we calculated the corresponding phase sensitivity $s_{1}\in [20,030,\;43,000]$ at $b\in [1.078946\times 10^{9},\;1.078948\times 10^{9}]$ and $\theta \in \left[49.432{}^{\circ},\;49.467{}^{\circ}\right]$, A total of $2.816\times 10^{5}$ groups of (b, θ, *s*) samples were used as the dataset, as shown in Figure 4b. This dataset not only covers the high-sensitivity parameter range but also includes parameter samples from partial transition regions. It can provide sufficient feature information for the training of deep learning models, thereby ensuring the model’s learning accuracy regarding the parameter-sensitivity mapping relationship. In the future, for SPR biosensors based on WSMs, the sensing characteristics of the two parameters—the Weyl node separation distance and the twist angle—within other value ranges can be explored.

**Figure 5:**
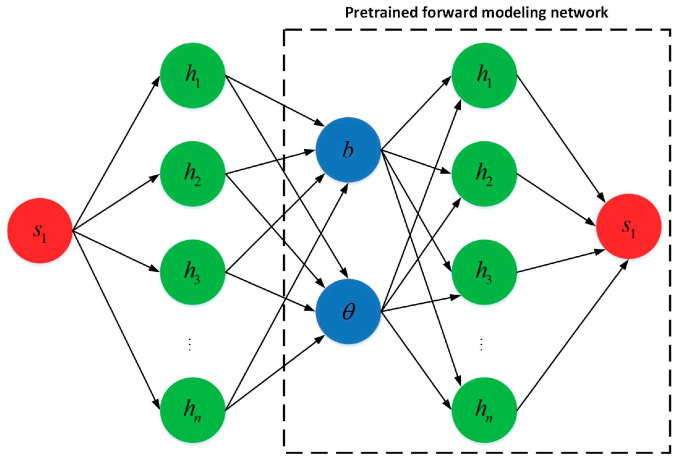
Reverse design neural network architecture for terahertz biosensors.

In order to further analyze and predict sensing performance, we also constructed an inverse design model for the THz biosensor with the structure shown in Figure 1, based on the Tandem Network Framework (TNF) [42]. This model consists of two components: a Forward Network and an Inverse Network. The Forward Network adopts a Fully Connected Neural Network (FCNN) to realize the nonlinear mapping from the Weyl node separation distance and the twist angle to the sensitivity $s_{1}$ with its architecture being 2−256−512−512−512−256−128−1. The Inverse Network also uses a fully connected structure, with an architecture of 1−512−256−256−256−2. Among them, the output of the Inverse Network serves as the input to the Forward Network, forming a tandem structure. As shown in Figure 5, the first neural network is identical to the traditional forward design network, while the second component is trained to predict the forward network corresponding to the design [42]. Both networks are illustrated in Figure 5. The tandem architecture trains the two networks in a cascaded manner. During the inverse phase, the weights of the Forward Network are frozen to ensure that after the inversely generated (b, θ) structures are mapped through the Forward Network, their outputs match the target sensitivity $s_{1}$ with high accuracy. In the context of inverse design, the ’multi-structure–single-response’ relationship implies that multiple geometric configurations may produce identical optical responses, thereby complicating network convergence. The proposed serial network framework mitigates this issue by effectively constraining non-uniqueness, ensuring a one-to-one mapping between device geometry and sensor performance. Data collection was completed via simulation using the TMM under the MATLAB(R2017a) parallel computing framework, generating a total of 200,000 groups of (b, θ) samples. The parameter ranges are as follows: $b\in [1.078946\times 10^{9},1.078948\times 10^{9}]$, $\theta \in \left[49.432{}^{\circ},49.467{}^{\circ}\right]$ and the corresponding sensitivity range is $s_{1}\in [20,030,\;43,000]$. The distribution of these samples is shown in Figure 4b. For network training, we split the aforementioned dataset into a training set and a test set at a ratio of 9:1. All input and output data were linearly normalized to intervals to accelerate network convergence and improve generalization ability. During the training process, the Mean Squared Error (MSE Loss) was adopted as the loss function, and the Adam optimizer was used to iteratively update the network weights. The initial learning rate was set $1\times 10^{-4}$. In addition, a cosine annealing strategy was integrated to dynamically adjust the learning rate. The optimization results show that after data normalization, the loss value of the Forward Network on the test set is $Loss_{1}=2.658\times 10^{-5}$. This fully demonstrates the high accuracy and stability of the constructed Forward Network in forward modeling tasks. And the test set loss value of the Inverse Network is $Loss_{2}=7.392\times 10^{-5}$.

**Figure 6:**
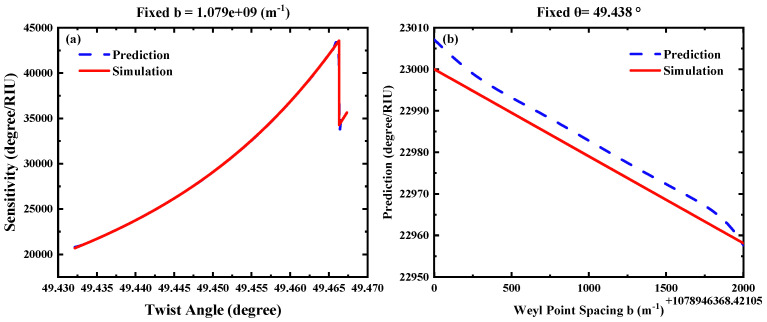
Predicted versus simulated values of sensitivity when Forward Network is fixed (**a**) the Weyl node separation distance and (**b**) twist angles of the WSMs at the median value, respectively.

To verify the performance of the Forward Network model, we conducted one-dimensional scans for each of the two free parameters at the midpoint of the dataset, as shown in Figure 6. When the Weyl node separation distance is fixed at the median value $b_{mid}=1.078948\times 10^{9}\;m^{-1}$, the network’s predicted results (red solid line) for the sensitivity $s_{1}$ as a function of the twist angle θ are in high agreement with the simulated values (blue dashed line), as illustrated in Figure 6a. When the twist angle θ is greater than 49.465°, the phase sensitivity decreases sharply. As shown in Figure 6b, under the twist angle $\theta =\theta_{mid}=49.4382$, the network’s predicted results (red line) for the b-scan also coincide well with the simulated values (blue dashed line). Subsequently, we validated the proposed inverse design framework against five randomly selected groups of target sensitivities $s_{1}$ (Case 1–5), as shown in Table 1. After solving for the corresponding structural parameters (b, θ) via the Inverse Network, the actual sensitivity $s_{1}$ obtained through simulation calculations. The above training results verify the effectiveness of the aforementioned mechanism: by training the forward modeling network and the inverse design network collaboratively in a tandem architecture, the challenge of non-unique mapping is effectively addressed [41]. This study provides a parameter optimization scheme for the inverse design of nanophotonic devices by deep learning.

## 4. Conclusions

To summarize, we have designed a terahertz high-sensitivity SPR phase biosensor with a KR structure based on WSMs. The combination of a coupling prism and WSMs to excite SPR provides a foundation for achieving higher phase sensitivity. Meanwhile, the Weyl node separation distance and the twist angle of WSMs offer means for realizing dynamic regulation. By adjusting the Weyl node separation distance and the twist angle of WSMs to achieve dynamic regulation, we systematically analyzed the sensor’s performance in the THz band. The results show that in gas sensing scenarios, by optimizing these two parameters, the phase detection sensitivity can reach 22,402 °/RIU. Compared with traditional SPR biosensors, the sensitivity is significantly improved. Moreover, we have further optimized the sensor structure and its parameters based on deep learning-enabled forward modeling and inverse design. We hold a belief that this scheme can provide a new and potentially applicable approach for fields such as chemical and biomolecular detection.

## Figures and Tables

**Table 1:** Validation of the proposed inverse design framework using five selected groups of sensitivity targets (Case 1–5).

Case	$\mathrm{Target}\;s_{1}$ (°/RIU)	$\mathrm{Design}\;s_{1}$ (°/RIU)	b ($m^{-1}$)	*θ* (°)
1	$3.62\times 10^{4}$	$3.6223\times 10^{4}$	1,078,947,449.4473	49.45931826
2	$3.65\times 10^{4}$	$2.6509\times 10^{4}$	1,078,947,278.8806	49.44560753
3	$4.02\times 10^{4}$	$4.0166\times 10^{4}$	1,078,947,592.2534	49.46330384
4	$2.30\times 10^{4}$	$2.3012\times 10^{4}$	1,078,947,355.0578	49.43827033
5	$3.30\times 10^{4}$	$3.3033\times 10^{4}$	1,078,947,403.2142	49.45556571

## Data Availability

The data presented in this study are available on request from the corresponding author.
